# Learning to Spell in Arabic: The Impact of Script-Specific Visual-Orthographic Features

**DOI:** 10.3389/fpsyg.2020.02059

**Published:** 2020-08-25

**Authors:** Rana Yassin, David L. Share, Yasmin Shalhoub-Awwad

**Affiliations:** Department of Learning Disabilities, Edmond J. Safra Brain Research Center for the Study of Learning Disabilities, University of Haifa, Haifa, Israel

**Keywords:** spelling, Arabic, development, orthography, writing systems

## Abstract

Learning to spell is a challenging process, especially for young learners, in part because it relies on multiple aspects of linguistic knowledge, primarily phonological and morphological. However, alongside these universals, there are significant writing system specifics, namely, language-specific and script-specific factors that may also challenge young readers and writers (Daniels and Share, 2018). The current study focuses on the impact of four distinctive visual-orthographic features of the Arabic abjad on spelling, namely, (i) the similarity of many basic letter-forms, (ii) allography (the positional variants of the letter forms), (iii) ligaturing (the joining of letters), and (iv) non-linearity (extra-linear diacritic-like signs used to mark consonantal, short vowel and morpho-syntactic distinctions). We examined the distribution of visual-orthographic spelling errors across three grade levels as well as the developmental changes in these errors. We predicted that these errors would account for a significant proportion of children’s spelling errors. Ninety-six Arabic-speaking pupils from three elementary grades (1st, 2nd, 4th grades) were presented with a sequence of six pictures and asked to write a story or several sentences about the events depicted. All spelling errors were analyzed and categorized according to two types of categories: six visual-orthographic categories and six additional categories that relate to the more traditional error types (e.g., phonological). The results showed that the visual-orthographic category was the second most common error category across the three grade levels, accounting for over one quarter of all spelling errors. Ligaturing and letter shape formation errors emerged as the two most prevalent types of errors in this category. These findings clearly demonstrate that visual-orthographic features of the Arabic abjad pose significant challenges in learning to spell.

## Introduction

To be literate today, an individual must not only be able to read but to write. Fluent written expression depends on a host of higher-order skills, but also on basic skills such as correct letter formation and the rapid and relatively effortless production of accurate word spellings. Learning to spell, however, is a complex and challenging process, especially for young learners because spelling typically relies on multiple aspects of linguistic knowledge, phonological, morphological and orthographic (Ehri, 1997; Perfetti et al., 1997; Bryant and Nunes, 2004; Nunes and Bryant, 2004; Treiman and Kessler, 2005; Verhoeven and Carlisle, 2006; Senechal and Kearnan, 2007; Ravid, 2012; Treiman, 2017; Perret and Olive, 2019).

Despite the impressive body of research findings on spelling development, the vast majority of this work has been undertaken in English, or in a few cases, other Western European (Roman) alphabets. Most children around the globe, however, learn to read and write in non-alphabetic writing systems such as Semitic abjads (e.g., Arabic and Hebrew), Brahmi-derived Indic abugidas, or morpho-syllabic Chinese. Relatively few studies have examined spelling development in these non-alphabetic scripts. The present study focuses on early spelling development in a non-European, non-alphabetic script – Arabic, a Semitic abjad.

Our approach to the study of spelling development is guided by the view that, alongside reading and spelling universals such as the representation of sound (phonology) and meaning (morphology), there are also significant writing system specifics, namely, language-specific and script-specific dimensions of writing system complexity that may also challenge young readers and writers (Perfetti and Harris, 2013; Daniels and Share, 2018). Ironically, the two most influential theoretical frameworks for describing cross-script diversity – Orthographic Depth (Katz and Frost, 1992) and Psycholinguistic Grain Size Theory (Ziegler and Goswami, 2005) give little consideration to non-European writing systems, both promoting a one-dimensional view of script variation, namely, spelling-sound (in)consistency. Daniels and Share (2018) have argued that theories of learning to read and write need to take into account the full range of writing system diversity. They propose that consideration of the full picture of the world’s writing systems reveals multiple dimensions of complexity and call for future research to investigate the impact of these dimensions on reading and spelling. The present investigation responds to this call by exploring the impact of Arabic’s unique visual-orthographic features on the early development of spelling. Our choice of Arabic is motivated by a number of factors.

First, Arabic is the sixth most spoken language in the world with close to 300 million speakers (Eberhard et al., 2019). It is the official language of 22 countries, and also the religious and liturgical language of more than 1.5 billion Muslims worldwide (Bokova, 2012). Second, the Arabic language has a unique orthography, containing a number of specific visual-orthographic features (common letter shapes, allography, ligaturing/cursivity, and non-linearity) all of which are pervasive in Arabic but rare or absent in most alphabetic scripts^1^. Shedding light on these special features is essential for a complete science of literacy learning. Third, a small but growing number of studies have begun to investigate the effect of these specific visual-orthographic features of the Arabic writing system on reading (e.g., Asaad and Eviatar, 2013; Dai et al., 2013; Ibrahim et al., 2013), but none has yet examined this issue in spelling.

### Arabic Orthography

Arabic is a Semitic language written in an *abjad* or consonantal writing system (Daniels, 1992, 2018; Saiegh-Haddad, 2018). Arabic script is fundamentally cursive and is written from right to left (Azzam, 1993; Saiegh-Haddad and Henkin-Roitfarb, 2014). Arabic orthography consists of two sets of graphic signs: horizontally arrayed letters and vertically arrayed extra-linear diacritic-like signs. Twenty-eight of the 29 letters denote consonants, and 2 letters (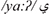 and 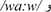) also represent the long vowels /*i:/* and /*u:/*, respectively. One more letter, 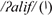 represents the long vowel /*a:/* (Bar-On et al., 2018).

The four main visual-orthographic features of Arabic are (i) the similarity of the basic letter-forms, (ii) allography (the positional variants of the letter forms) (iii) ligaturing (the joining of letters), and (iv) non-linearity (the use of extra-linear diacritic-like signs to mark consonantal, short vowel and morpho-syntactic distinctions).

***(i) The similarity of many basic letter forms****:* One of the characteristic features of Arabic orthography is the similarity of many basic letter-forms. This feature stems from the fact that Arabic contains many more consonants than the Nabatean script from which it was derived (Daniels, 2018). A majority of letters have an identical or near-identical structure and are distinguished only by the existence, placement, and the number of dots (Eviatar and Ibrahim, 2014). These dots are non-optional and are considered an integral part of a letter, as in the case of the dot in the English lowercase letters < i > and < j >. Seven pairs of letters (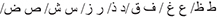) and two triplets (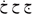) and (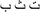) share the identical letter shape (*rasm*) (the complete inventory of Arabic letters is shown in Appendix A).

***(ii) Allography****:* Another pervasive characteristic of Arabic orthography is allography, namely, the variability of letter forms. This variability depends on two factors. First, its position in the word – initial, medial, final. Second, whether or not it connects to the letter that precedes it. Together, letter position and ligaturing create the allographic variants: 23 letters are considered to have four letter-forms, and six letters have two forms (see (iii) Ligaturing/Cursivity). For example, the letter 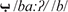 is written 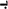 when in initial position as well as in medial position when not ligatured to the previous letter, in medial position and ligatured to the previous letter 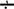, and in word-final position 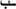, and 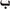 (ligatured and unligatured respectively). Note that both word-final forms have the characteristic word-final flourish or “tail” (see Appendix A). This highlights the fact that not all allographic variants are the product of ligaturing (as discussed in (iii) Ligaturing/Cursivity). In addition to the word-final flourish, there are internal form changes in the final vs. non-final 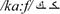, the open loops of the word-final */mi:m/* 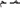, and minor changes in the location of the dotting in several letters (e.g., 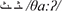). Two studies have now demonstrated that positional variants of letters affect word reading in Arabic (Taouka and Coltheart, 2004; Asaad and Eviatar, 2014). Both studies reported that incorrect positional variants (such as a word-final letter appearing in the middle of a word) slow reading times and reduce reading accuracy.

***(iii) Ligaturing/Cursivity****:* Cursivity is perhaps the most conspicuous feature of Arabic (Saiegh-Haddad and Henkin-Roitfarb, 2014; Yakup et al., 2015). The majority of the letters in a word connect to the adjacent letters creating a word that forms a single unbroken graphic unit. Thus, three types of words are possible: a fully connected word 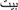 /*bayt/ “home,”* a partly connected word 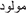 /*mawlu:d/* “born” and an entirely unconnected word 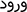 /*wuru:d/* “roses.” All 29 letters can connect to the previous letter (on the right side). For example, the letter named 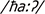 (letter sound 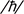) can be connected on the right or the left 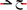 or on both sides 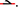 Similarly, the letter 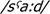 (letter sound 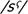) can also be right-connected or left-connected 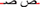 as well as doubly connected 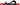 and so on. In word formation, these two letters 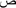 and 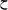 are joined as 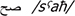 “correct.” Six letters, however, have only two variant forms which can connect only from the right but not the left side (e.g., 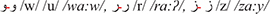, 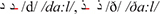, and 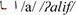). For example, the letter 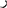 /*r*/ is unconnected in 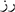 */ruz/* “rice,” but (right-) connected in 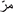 /*murr/* “sour” (Saiegh-Haddad and Henkin-Roitfarb, 2014). Although some letter variants in Arabic involve systematic alterations (principally additions) depending on position (e.g., 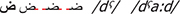 and 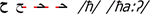), some changes are substantial to the point of appearing quite unrelated (e.g., 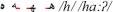).

***(iv) Tashkeel and Non-linearity****:* An additional feature of Arabic orthography is the extensive use of extra-lineal diacritic-like signs. These marks are placed mostly above but also below letters unlike the letters of most alphabets which are arrayed along a single horizontal axis. Daniels and Share (2018) refer to this dimension as non-linearity. In addition to the consonant dots discussed above in (i), Arabic orthography includes two classes of extra-lineal signs, named tashkeel: phonemic and morpho-syntactic (Saiegh-Haddad, 2018). The phonemic tashkeel consist of five major marks, three of which consistently map the three short vowels; 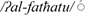 represents 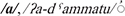 represents /*u/*, and 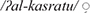 represents /*i*/, one that denotes vowel nullification (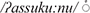), and one that denotes consonant gemination (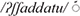) (N.B. the broken circle represents any consonant letter). The phonemic tashkeel can appear on almost any letter within the word, and they map contrastive phonemic information. In contrast, the three short vowels (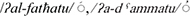, and 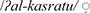) can also appear word-finally, in which case they map morpho-syntactic properties such as noun case and verb mood (Saiegh-Haddad, 2018). Finally, there are another three extra-lineal signs, called nunation /*tanwi:n*/, which also have a morpho-syntactic function (i.e., the case endings of indefinite nouns) and only appear word-finally. They consist of the three vowel signs doubled to indicate that the vowel sound is followed by the consonant /*n***/**: double *fat*ℏ*a* (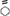) /an/, double 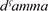 (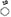) /un/, and double *kasra* (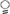) /in/ (Saiegh-Haddad, 2018). The tashkeel also includes the following less frequent signs: /*madda*/~, 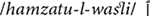 which only appears on the alif, and the “dagger” alif or superscript alif 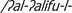 *xanjariyyatu*/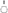 (see, for details, Saiegh-Haddad and Henkin-Roitfarb, 2014). The presence of tashkeel in Arabic text is called mashkul script, and makes the orthography phonologically transparent. The mashkul script is primarily used for early reading instruction at the onset of formal schooling, from first to fourth grades, and in children’s books. It is also used for the Quraa’n and in poetry (Bar-On et al., 2018). However, the second version of Arabic script, the default for Arabic speakers, is the non-mashkul script /*γayr-mashku:l/*, which relies on letters alone with no tashkeel other than the non-optional consonant dotting. The non-mashkul Arabic script is often considered deep because words can potentially be assigned many phonological forms, corresponding to both lexical and non-lexical readings (Bar-On et al., 2018; Saiegh-Haddad, 2018).

### Studies of Spelling Development in Arabic

Understandably, studies of literacy learning in Arabic (and Hebrew) have mainly focused on the role of phonology. Indeed, several investigations have shown that phonological awareness plays a crucial role in spelling development in Arabic (Abu-Rabia and Taha, 2006; Batnini and Uno, 2015; Saiegh-Haddad and Taha, 2017). In a series of studies of spelling development, Abu Rabia and colleagues found that the most common type of spelling error committed by native Arabic-speaking children (and especially dyslexics) is phonological (Abu-Rabia and Taha, 2004, 2006; Abu-Rabia and Sammour, 2013). For example, Abu-Rabia and Sammour (2013) reported that most of the spelling errors occurred as a result of confusing emphatic consonants and their non-emphatic counterparts (e.g., 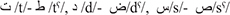, and 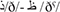). In addition to studies emphasizing phonology, several recent studies have emphasized the role of morphology in the early stages of Arabic spelling (e.g., Taha and Saiegh-Haddad, 2016; Saiegh-Haddad and Taha, 2017; see also Ravid, 2012). In this context, it is important to note that Semitic languages such as Arabic have a dense morphological structure as most content words (all verbs as well as most nouns and adjectives) are made up of two independent and unpronounceable bound morphemes: a root and a word pattern. The root is a consonantal skeleton that provides the word’s core meaning, and the word pattern is a fixed prosodic template that specifies the word’s categorical meaning and some of the phonological characteristics of the surface form (vocalic, syllabic, and prosodic form) (McCarthy, 1981; Saiegh-Haddad and Henkin-Roitfarb, 2014; Boudelaa and Marslen-Wilson, 2015; Shalhoub-Awwad and Leikin, 2016). The root-and-pattern structure of Arabic is also a salient feature of the orthographic structure of written Arabic (Saiegh-Haddad and Taha, 2017). Research has shown that elementary school children in second, fourth and sixth grades use derivational morphological structure to spell real and pseudowords (Taha and Saiegh-Haddad, 2017). Furthermore, morphological awareness has been found to predict unique variance in spelling development, beyond phonological awareness and general cognitive skills among normal and reading-disabled children (Saiegh-Haddad and Taha, 2017). It has also been shown that both morphological and phonological interventions have a significant impact on spelling in Arabic, among normal and reading-disabled children, especially in the initial grades (Taha and Saiegh-Haddad, 2016).

To date, only a single study has focused on the challenges that beginning spellers incur due to the unique visual-orthographic features of the Arabic writing system. Dai et al. (2013) examined the influence of letter ligaturing on printed word learning (“orthographic learning”) in third-grade Arabic readers. Test stimuli consisted of forty pseudowords, all with the *fatħa* short vowel sign. Half of the pseudowords consisted of non-connecting letters (e.g., 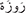) whereas the other 20 pseudowords were composed of the remaining letters of the Arabic orthography which were all presented in their connecting form (e.g., 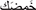). The children were asked to read aloud and to spell all test stimuli (20 connected and 20 unconnected items). The results showed that connectedness not only slowed down reading speed but also reduced accuracy for spelling.

As mentioned above, the greater part of the current literature on spelling development in Arabic has focused on the contributions of universal factors such as phonology and morphology, rather than the script-specific visual-orthographic dimensions of the Arabic writing system. The main purpose of the current study is to examine the effect of the four distinctive script-specific visual-orthographic features of the Arabic writing system reviewed above (similarity of basic letter-forms, allography, ligaturing, and non-linearity) on spelling among children in Grades 1, 2, and 4. We hypothesized that visual-orthographic factors of Arabic orthography would account for a non-trivial proportion of spelling difficulties.

## Materials and Methods

### Participants

Ninety-six pupils from three grades participated in this study: 32 first graders, 32 second graders, and 32 fourth graders^2^ (16 boys and 16 girls from each grade). Children were recruited from four Arabic-speaking elementary schools in the north of Israel. These schools were selected to represent a wide range of socio-economic backgrounds and included two schools from a middle SES neighborhood, a third high-SES school, and fourth low-SES school. All participants were native speakers of the local dialect of Palestinian Arabic spoken in the north of Israel. Complete classrooms were tested in a group-testing situation with no child excluded.

### Materials

The Picture Story Writing Task (adapted from Mayer, 1969) was administered to all participants. In this task, each child was presented with a sequence of six color pictures (see Appendix B) and asked to write a story or several sentences about the events depicted in the pictures. These six pictures were taken from the wordless picture book, *Frog, where are you?* (Mayer, 1969). The booklet, consisting of 25 pictures, depicts the adventures of a child and his puppy, out to search for a frog that has gone missing. This writing task has been used extensively in cross-linguistic work among a wide range of age groups (Berman and Slobin, 1994). It contains no words and provides a fairly rich context for spoken and written language production (Reilly et al., 2004).

It should be noted that the abbreviated six-picture version that we administered omits some of the attempts to search for the lost frog but keeps the main plot of the story events intact. Similar to other research on story writing (e.g., Berninger et al., 1997; Graham et al., 2000), the choice of this particular task was solely designed as a trigger to elicit writing, tailored especially for first and second graders who are used to perform reading and writing tasks involving pictures. The written productions were used to generate a naturalistic corpus of spelling errors.

### Procedure

The task was administered toward the end of the school year to entire first, second, and fourth-grade classrooms in the four schools. In each of the four schools, two classes in each grade level were randomly selected. Instructions were given in the pupils’ mother tongue to the entire class. Each student in the class received the set of pictures, an empty ruled page, and the following instructions: “*Look at the pictures in the correct order (each picture was numbered from 1 to 6) and write a story or some sentences telling the story of what is happening in the pictures.*” The task was administered in a single 45-min lesson.

### Error Analysis

Our error analysis took into account the fact that children’s written productions across this range of ages vary considerably in terms of lexical, morphological and orthographic content and complexity. Because error types may also vary as a consequence of these differences, we confined our error analysis to a subset of 45 key words common to all productions across the three grade levels (first, second, and fourth grade). A subset of 45 words were selected after examining the pupils’ productions and then selecting those words that appeared most frequently across all three grade levels. This corpus included 37 content words – 19 nouns (e.g., 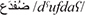 ‘frog’), 18 verbs (e.g., 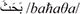 “looked for”), and 8 function words (e.g., 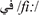 “in”).

The total number of words in each pupil’s written production was counted, along with the number of words from the corpus of 45 words. If a child wrote a word more than once, each production was counted separately because this created an opportunity for an error. Spelling errors were then recorded and classified according to two types of categories: six categories relevant to the visual-orthographic focus of the present study and six additional categories that relate to the more traditional error types (e.g., phonological). The first four visual-orthographic categories were based on four of the 10 dimensions of orthographic complexity discussed by Daniels and Share (2018), namely, *letter-form confusion* (errors caused by confusion between letters with the same or very similar structure which are differentiated only by the existence, placement, and number of dots), *allography* (errors caused by selecting the inappropriate positional variant of a letter form (word-initial, medial or final), *ligaturing* (an inappropriate connection or omission of an obligatory connection between adjacent letters), and *non-linearity* (errors in the location and/or order of the extra-lineal signs (tashkeel and letter dotting) in the vertical axis). Most fourth graders (who typically are used to non-mashkul script) did not write with Arabic’s optional tashkeel, therefore, we decided to ignore this dimension for all pupils. This decision stemmed from the fact that when administering the writing task, no direct instruction was given to the pupils to write with tashkeel, since our purpose was to obtain a naturalistic corpus of errors. A fifth category **—** *letter shape formation*, emerged in the course of data coding. This category comprised errors in which the child either added or omitted an integral feature of letter form. The sixth category included *other* (unclassifiable) visual orthographic errors such as illegible productions (see Table 1). The six additional (*non*-visual-orthographic) error categories were *phonological* (errors caused by inappropriate application of sound-symbol correspondence rules that change the phonemic makeup of a word resulting in an incorrect pronunciation, e.g., 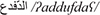 instead of 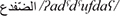 “the frog”), *spelling conventions* (caused by incomplete mastery of spelling conventions, e.g., 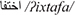 instead of 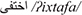 “disappeared,” which is phonologically permissible but is the wrong form of the letter 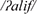 (i.e., 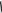 instead of 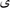)), *morpho-orthographic* (errors caused due to lack of reliance on suitable word-pattern or clitics, e.g., 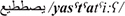 instead of 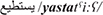 “can,” this error occurred due to a lack of reliance on the appropriate verbal word-pattern letters), *morpho-syntactic* (errors caused by inappropriate application of vocalic word endings which denote the syntactic categories of case and mood, e.g., 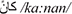 instead of 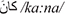), *diglossic* (errors that transcribe the spoken or colloquial form instead of the standard Arabic form, e.g., 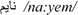 instead of 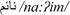), and a category of *other* error types (unclassifiable), e.g., errors caused by adding two graphic signs to a letter which represent two short vowels.

**TABLE 1:** Examples of spelling errors from the six visual-orthographic categories.

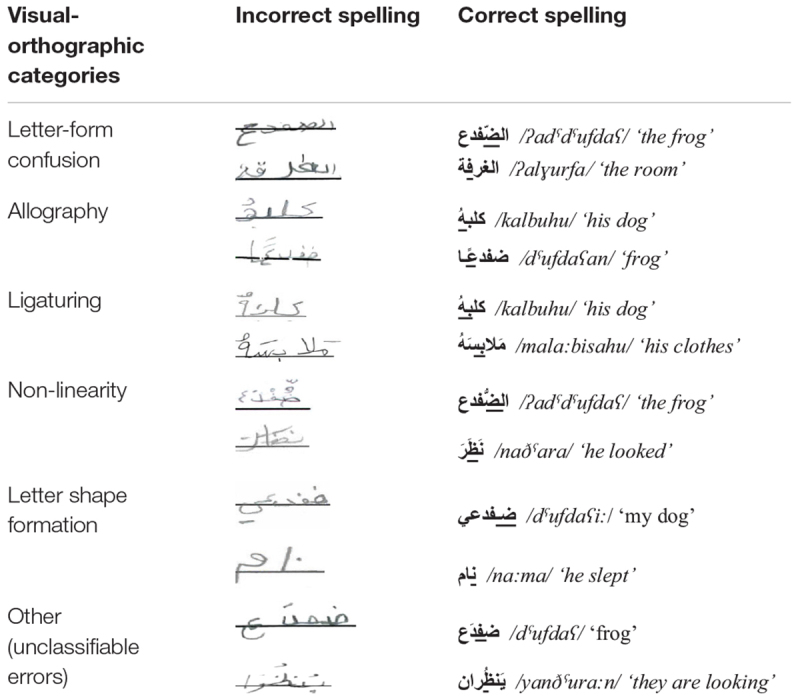

*Some of the incorrect spellings contain errors from the six additional (non-visual-orthographic) categories.*

## Results

Table 2 presents the total number of words written by the children across the three grades, the number of words in the selected corpus of 45 key words, the proportion of corpus words out of the total number of words produced, the total number of spelling errors in this corpus, and the error rate per word. Table 2 shows that, with increasing grade levels, children, as expected, wrote more words, and spelled these words more accurately. Significant differences across grades were found in the proportion of corpus words out of the total number of words produced [*F*(2, 93) = 18.07, *p* < 0.001, η*_*p*_*^2^ = 0.28]. Bonferroni *post-hoc* contrasts revealed a significantly larger proportion of corpus words in Grade 1 compared to Grade 2 (*p* < 0.001), and to Grade 4 (*p* < 0.001), but no significant difference between grades 2 and grade 4 (n.s.) (see Table 2). As for the spelling errors within the 45 corpus words, children committed around two errors per three words in both Grades 1 and 2, and even after 4 years of formal schooling, children were still far from achieving spelling mastery, making on average one error in every second word. This confirms that spelling is indeed a challenging task in Arabic.

**TABLE 2:** Means and standard deviations (in parentheses) of the total number of words produced per child, the mean number of words in the selected corpus of 45 key words, the proportion of corpus words out of the total number of words produced, the mean number of spelling errors in the 45-word corpus, and the error rate per word across three grades.

	Grade 1	Grade 2	Grade 4
Total words produced	43.1 (9.67)	64.6 (20.55)	82.9 (32.43)
Mean number of corpus words	29.1 (6.34)	34.9 (9.73)	46.1 (14.98)
Proportion of corpus words out of the total number of words produced (in percentages)	68.6 (11.47)	55.0 (7.36)	57.5 (9.65)
Total spelling errors (in the 45-word corpus)	20.7 (10.39)	22.2 (10.78)	22.7 (14.64)
Error rate per word	0.71 (0.32)	0.68 (0.37)	0.48 (0.25)

The first aim of this study was to determine the overall proportion of visual-orthographic spelling errors. We predicted that this category of errors would account for a non-trivial proportion of children’s errors. This category combined the four dimensions of ligaturing, letter shape confusion, allography and non-linearity, as well as the newly added category of letter shape formation. The data presented in Table 3 show that the visual-orthographic category was the second most common error category across the three grade levels, accounting for over one quarter (27.2%) of all spelling errors. This finding clearly shows that visual-orthographic errors, as anticipated, constitute a significant proportion of young children’s naturally occurring spelling errors. Turning to the more traditional categories of *non*-visual-orthographic errors, the most common errors were violations of *spelling conventions*, comprising between one third and one half of all errors. The *phonological* category was the third most prevalent category accounting for around one quarter of all spelling errors. It is important to note, however, that the category of phonological errors, which has received the most attention in the Anglophone literature was eclipsed by the (combined) visual-orthographic category that was the particular focus of the present investigation. Each of the three remaining non-visual-orthographic categories – *morpho-orthographic*, *morpho-syntactic*, and *diglossic*, each accounted for only a few percent of the total corpus of errors (see Table 3). One-way between-subjects ANOVA were conducted to examine developmental changes across grades in the proportion of errors in the combined visual-orthographic category and the six additional categories. Significant differences across grades were evident in the proportion of visual-orthographic spelling errors [*F*(2, 93) = 8.06, *p* = 0.001]. Bonferroni *post-hoc* contrasts revealed significantly greater visual-orthographic error rates in Grade 4 compared to Grade 1 (*p* < 0.001), but no significant difference between grades 1 and grade 2 (*p* = 0.108), or between grades 2 and 4 (*p* = 0.188) (see Table 3). Regarding the six additional categories, there was a significant decline in the proportion of spelling convention errors [*F*(2, 93) = 8.93, *p* < 0.001] but the proportion of phonological errors remained steady across the grades. This latter finding indicates that phonology continues to trouble young spellers throughout most of their elementary years. There was also a significant but small decrease (amounting to a few percentage points) in morpho-orthographic errors. No other developmental differences were evident in the other non-visual-orthographic categories.

**TABLE 3:** Means (in percentages) and standard deviations (in parentheses) of error rates for the combined visual-orthographic category and the six additional categories at three grade levels.

Error category	Grade 1	Grade 2	Grade 4	Total	*P*	Effect Size (η*_*p*_*^2^)
	%	%	%	%		
**Visual-orthographic (combined)**						
	18.2 (13.91)	27.5 (16.62)	35.8 (21.23)	27.2	<0.01**	0.148
**Non-visual-orthographic**						
Phonological	18.4 (14.15)	23.9 (17.50)	27.7 (23.01)	23.3	0.137	0.042
Spelling conventions	52.3 (19.04)	41.9 (17.00)	32.6 (19.74)	42.3	<0.001***	0.161
Morpho-orthographic	5.8 (6.63)	3.6 (4.89)	1.9 (3.97)	3.8	<0.05*	0.085
Morpho-syntactic	2 (4)	1.1 (2.80)	1 (2.22)	1.4	0.328	0.024
Diglossic	2.8 (4.83)	1.2 (2.21)	0.8 (3.45)	1.6	0.082	0.052
Other	0.5 (1.66)	0.8 (2.55)	0.2 (0.98)	0.5	0.406	0.019
Total	100	100	100	100		

*p < 0.05, **p < 0.01, ***p < 0.001.

The second aim of the current study was to examine the distribution of the visual-orthographic spelling error categories as well as the developmental changes that occur in these errors across the three grade levels. Table 4 displays the error rates for the separate visual-orthographic categories that were the main focus of the present study. The results showed that both ligaturing errors and letter shape formation were the two most prevalent types of errors, each accounting for around one third of all visual-orthographic errors across grades. Confusion of identical or near-identical letter forms (10.7%) and allographic substitutions (8.4%) also contributed a non-trivial number of visual-orthographic errors. The *non-linearity* category, for reasons already discussed in the method section, comprised less than 1% of all visual-orthographic errors (see Table 4).

**TABLE 4:** Means (in percentages) and standard deviations (in parentheses) of error rates for the separate visual-orthographic categories across three grade levels.

Error types	Grade 1	Grade 2	Grade 4	Total	*P*	Effect size (η*_*p*_*^2^)
	%	%	%	%		
Letter-form confusion	6.5 (20.05)	13.3 (25.11)	12.2 (23.79)	10.7	0.456	0.017
Allography	13.5 (25.14)	5.1 (15.43)	6.6 (15.12)	8.4	0.179	0.036
Ligaturing	22.1 (26.81)	46.5 (41.23)	38.2 (37.46)	35.6	<0.05*	0.077
Non-linearity	1.6 (8.84)	0.9 (3.63)	0 (0)	0.8	0.525	0.014
Letter shape formation	34.6 (38.48)	30.6 (37.48)	35.9 (35.26)	33.7	0.836	0.004
Other	6.1 (20.14)	0.4 (1.78)	0.8 (2.36)	2.4	0.103	0.048

*Eight children from the sample did not make any visual orthographic errors: 5 first graders; 1 second grader; and 2 fourth graders. Therefore, as can be seen in the table, the sum of the error rates of all of the visual-orthographic categories for each grade does not total 100%.* *p < 0.05.

One-way ANOVAs were again used to examine developmental changes in each of the six visual-orthographic categories across the three grade levels. The only category with significant developmental change was *ligaturing* [*F*(2, 93) = 3.89, *p* = 0.024]. Bonferroni *post-hoc* contrasts indicated that, counter-intuitively, the proportion of *ligaturing* errors *increased* significantly from Grade 1 to Grade 2 (*p* < 0.05) then remained steady from Grade 2 to Grade 4 (*p* > 0.05) (see Table 4).

## Discussion

This study investigated the impact of Arabic’s unique visual-orthographic features on the early development of spelling. Participants were asked to compose a story based on six pictures taken from the wordless picture book, *Frog, where are you?* (Mayer, 1969). Spelling errors in a subset of 45 keywords common to almost all productions across three grade levels were recorded and classified into two types of categories: six categories relevant to the visual-orthographic focus of the present study and six additional categories that relate to the more traditional error types (e.g., phonological).

The findings revealed a high rate of errors across all grades. These findings are in line with an ample body of research that has been undertaken in English, and other (Western) European alphabets on spelling development showing that spelling is a complex and challenging process (Ehri, 1995; Perfetti et al., 1997; Bryant and Nunes, 2004; Nunes and Bryant, 2004; Treiman and Kessler, 2005; Verhoeven and Carlisle, 2006; Senechal and Kearnan, 2007; Ravid, 2012; Treiman, 2017; Perret and Olive, 2019). This study extended this conclusion to Arabic (a non-alphabetic script). Even after 4 years of formal schooling, children are still far from achieving spelling mastery.

The findings across the three grade levels regarding the proportion of visual-orthographic spelling errors relative to the six additional categories (phonological, spelling conventions, morpho-orthographic, morpho-syntactic, diglossic, and other) revealed that the *visual-orthographic category* ranked the second most frequent category, accounting for over one quarter (27.2%) of all spelling errors. This substantial proportion supports the view that there are significant script-specific dimensions of writing system complexity that pose obstacles for young spellers, alongside spelling (and reading) universals such as the representation of sound (phonology) and meaning (morphology) (Perfetti and Harris, 2013; Daniels and Share, 2018). Turning to the more traditional categories of *non*-visual-orthographic errors, the most common errors were violations of *spelling conventions*, comprising between one third and one-half of all errors. The *phonological* category was the third most prevalent error category accounting for around one quarter of all spelling errors. An unexpected outcome was the fact that errors in the combined visual-orthographic category were more prevalent than phonological errors, and continues to trouble young Arabic-speaking spellers throughout most of their elementary years. This finding diverges from the Anglophone emphasis on the phonological category as the most important and common category of spelling errors (Frith, 1985; Ehri, 1989; Nunes et al., 1997). It is also inconsistent with previous studies of Arabic spelling development which also concluded that phonological errors are the most common category of spelling errors committed by native Arabic-speaking children (Abu-Rabia and Taha, 2004, 2006; Abu-Rabia and Sammour, 2013). This inconsistency may stem from the fact that these studies largely ignored the unique visual-orthographic features of Arabic. For example, Abu-Rabia and Taha (2006) examined the spelling errors of Arabic-speaking children in the first through ninth grades. In addition to the conventional phonological categories, their reference to the visual-orthographic category was limited to the similarity between letter forms alone. There was no reference to the other specific visual-orthographic features of Arabic that were the special focus of the present study such as ligaturing, allography, or non-linearity. Another factor that may explain the inconsistency in the frequency of phonological errors in this study’s results vs. those of previous studies (such as Abu-Rabia and Taha, 2004, 2006; Abu-Rabia and Sammour, 2013,) is the difference in the way the errors were categorized, specifically the classification of morphological spelling errors as phonological errors. For example, in our study, when a pupil wrote the word 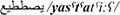 instead of 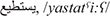, “can,” the errors were classified as morpho-orthographic, because they occurred due to a lack of reliance on the appropriate verbal word-pattern letters (see “Error Analysis” in the “Materials and Methods” section). In contrast, previous studies classified spelling errors of this type as phonological, as the phonological similarity between the emphatic consonants and their non-emphatic counterpart (e.g., 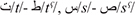), as well as the emphatic consonants, affect the boundary and/or neighboring syllables (This phenomenon of “velarization spread” is discussed in detail by Saiegh-Haddad, 2013). Hence, it is not surprising that their results showed that phonological spelling errors predominated over other error categories.

Within the broad category of visual-orthographic spelling errors, the most common error types were ligaturing errors and letter shape formation, each accounting for around one-third of all visual-orthographic errors across grades. The letter shape formation category had not been planned prior to conducting this study but emerged in the course of data coding. This category involving the addition or omission of an integral feature of letter shape (mainly the existence or absence of a small horizontal line, see examples in Table 1) was not discussed by Daniels and Share (2018) who focused on reading rather than writing. This type of error testifies to another dimension of difficulty (production difficulties) faced by the children in learning to spell. This error may also be a product of the high degree of similarity between many of Arabic’s cursive letters. This high rate of shape formation errors may also be related to the fact that at the onset of literacy acquisition, teachers may not emphasize the importance of adding this small horizontal line to letters. An unexpected finding was the significant *increase* in the error rate across age in the combined visual-orthographic category along with the developmental change in *ligaturing* errors which also *increased* significantly from first to second grade. We suspect that this counter-intuitive increase may stem from the fact that the task was a written expression task – focused on meaning-making. None of the instructions mentioned the issues we addressed in our study, or even the subject of spelling or handwriting legibility. It seems possible, therefore, that in the higher grades, children invested more effort in the content of their productions than in the mechanics of writing, leading to an increase in error rates. However, in first grade, at the onset of formal instruction in reading and writing, instruction focuses on learning the principles of the Arabic writing system. Thus, it is reasonable to assume that the younger children placed greater emphasis on the “mechanics” and “technical” or non-meaning-making aspects of writing than the older children. If this is correct, this would parallel the initial instructional emphasis on the “mechanics” of decoding or the “technical” side of reading in Israeli reading instruction when children are first introduced to reading and writing. We predict that in a traditional “pure” spelling dictation task, the older children would give greater attention to “mechanics” and produce significantly lower rates of ligaturing.

Our study represents a first foray into dimensions of writing system complexity that have, understandably, been largely ignored in research on spelling in English and other Western European alphabets which have primarily focused on the issue of phonology, and, to a lesser extent, morphology. Arabic is often described as a unique writing system, but many of the visual-orthographic features that are pervasive in Arabic, can be found, in varying degrees, in many of the world’s writing systems (Daniels and Bright, 1996), particularly the non-alphabetic systems which constitute a majority of the world’s scripts. Thus, the present investigation is not merely an investigation of an exotic or exceptional script, but represents one of the first steps (see also Nag et al., 2014; Chang et al., 2016) toward understanding dimensions of writing system complexity that have largely been ignored until now. Furthermore, the present findings are clear that these additional (visual-orthographic) dimensions have non-trivial ramifications for acquiring basic skills that are essential for competent written language production.

### Implications, Limitations, and Future Directions

The main pedagogical implication that may follow our findings is that more importance should be allocated to the instruction of the visual-orthographic aspects of the Arabic writing system that children find difficult (mainly ligaturing and shape formation) from the onset of literacy acquisition. This implication is supported by Treiman’s (1993) conclusion that children can master and gain knowledge of the easy aspects on their own, but they need direct instruction regarding difficult aspects that they struggle with.

As indicated earlier, the present investigation is one of the first steps toward understanding the impact of Arabic’s unique visual-orthographic features on the early development of spelling, so our emphasis was on examining the proportion of visual-orthographic spelling errors and their development across the three grades. We found that these errors constitute a significant proportion of children’s spelling errors at least from Grades 1 to Grade 4 and possibly beyond. However, this study did not address other important factors involved in spelling, such as handwriting (Graham, 1999; Graham et al., 2002). Future studies should examine the role of handwriting and kinesthetic-motor skills in this class of spelling errors. Another issue which merits pursuing is the impact of these visual-orthographic spelling errors on the quality of reading and writing.

We also need to acknowledge that our study was based on only a single sample of children’s naturalistic written productions. Future research will need to establish whether our results can be generalized to other genres of written productions. It is possible that certain types of errors – such as morphological or morpho-syntactic errors could be influenced by text genre (e.g., narrative vs. expository) but we see no reason to believe that the visual-orthographic errors which were the focus of this study would differ across genres.

## Data Availability Statement

The datasets generated for this study are available on request to the corresponding author.

## Ethics Statement

This study was approved by the Office of the Chief Scientist – Ministry of Education (permit #9667) as well as by the Research Ethics Committee of the Department of Education at the University of Haifa. Written informed consent to participate in this study was provided by the participants’ legal guardian/next of kin.

## Author Contributions

DS conceived the study. RY recruited the children and collected the data. RY and YS-A coded and analyzed the data. RY, YS-A, and DS wrote the manuscript. All authors contributed to the article and approved the submitted version.

## Conflict of Interest

The authors declare that the research was conducted in the absence of any commercial or financial relationships that could be construed as a potential conflict of interest.

## Footnotes

1 Ligaturing is the norm in Syriac, N’ko, Manchu, and Mongolian, whereas consonants in Brahmi-derived Indic scripts are often combined either horizontally or vertically via ligaturing. Non-linear diacritics are found, in varying degrees, in almost all European alphabets (e.g., Spanish, Czech, Polish) as well as non-European alphabets (e.g., Vietnamese, Thaana). Allography (e.g., upper-case and lower-case letters) is also common in many alphabets, at least in word-initial position. The phenomenon of common letter shapes (e.g., p, q, b, d) is well-known in English.

2 Third graders were not recruited for this study due to the limited resources at our disposal, both in terms of budget allotted and time constraints (particularly the need to gather the data for each grade level during the same time period at the end of the school year). Due to these constraints, recruiting fourth graders was prioritized (and not third graders), as the transition from mashkul script to non-mashkul script occurs in the fourth grade (see Bar-On et al., 2018).
