# Supplementary material for: Learning to Spell in Arabic: The Impact of Script-Specific Visual-Orthographic Features
Source: Front Psychol. 2020 Aug 25;11:2059. doi: 10.3389/fpsyg.2020.02059 (PMC7497809; doi:10.3389/fpsyg.2020.02059)
Supplement: Supplementary file 1 [file Data_Sheet_1.pdf]

Appendix A

Arabic letter forms

Final non ligatured	Final ligatured	Medial ligatured	Initial (or medial nonligatured)	IPA
ا	ـا	ـا	ا	a
ب	ـب	ـبـ	بـ	b
ت/ة	ـت/ـة	ـتـ	تـ	t
ث	ـث	ـثـ	ثـ	θ
ج	ـج	ـجـ	جـ	dʒ/g
ح	ـح	ـحـ	حـ	ħ
خ	ـخ	ـخـ	خـ	x
د	ـد	ـد	د	d
ذ	ـذ	ـذ	ذ	ð
ر	ـر	ـر	ر	r
ز	ـز	ـز	ز	z
س	ـس	ـسـ	سـ	s
ش	ـش	ـشـ	شـ	ʃ
ص	ـص	ـصـ	صـ	sˤ
ض	ـض	ـضـ	ضـ	dˤ
ط	ـط	ـطـ	طـ	tˤ
ظ	ـظ	ـظـ	ظـ	ðˤ
ع	ـع	ـعـ	عـ	ʕ
غ	ـغ	ـغـ	غـ	ɣ
ف	ـف	ـفـ	فـ	f
ق	ـق	ـقـ	قـ	q
ك	ـك	ـكـ	كـ	k
ل	ـل	ـلـ	لـ	l
م	ـم	ـمـ	مـ	m
ن	ـن	ـنـ	نـ	n
ه	ـه	ـهـ	هـ	h
و	ـو	ـو	و	w/u
ي	ـي	ـيـ	يـ	y/i

Appendix B

Picture prompts for the writing task

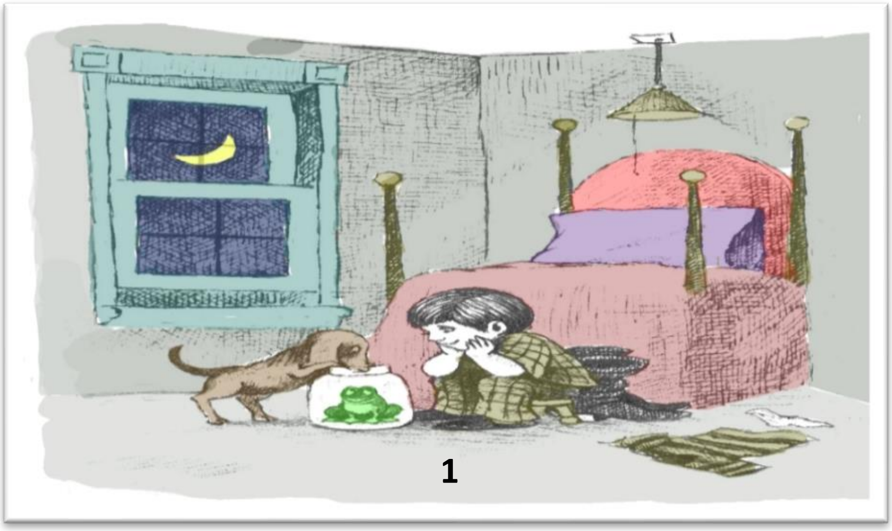

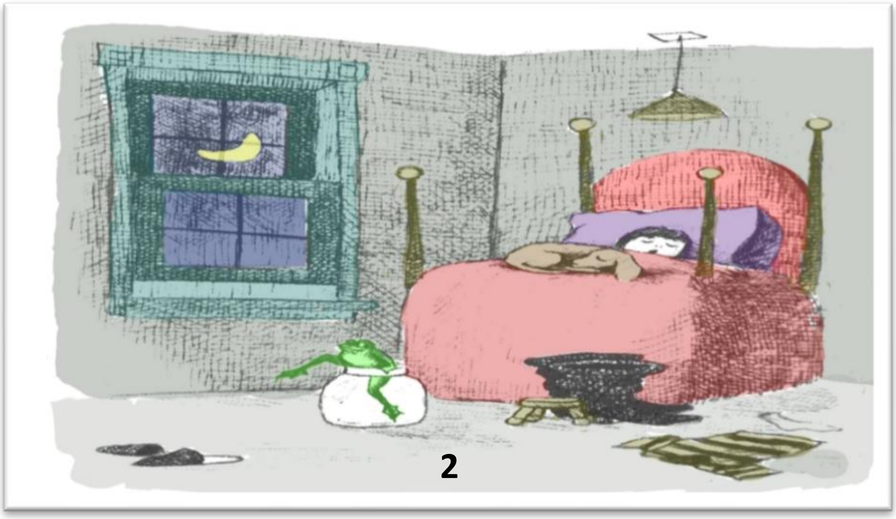

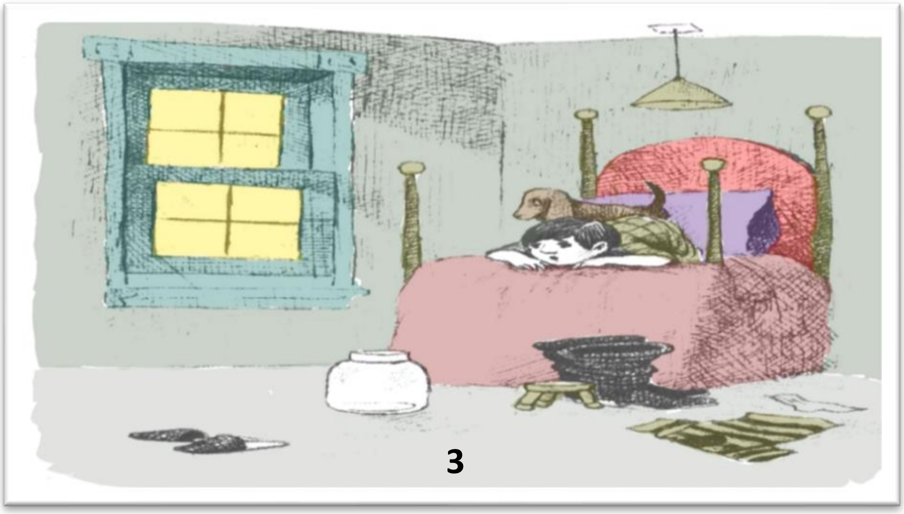

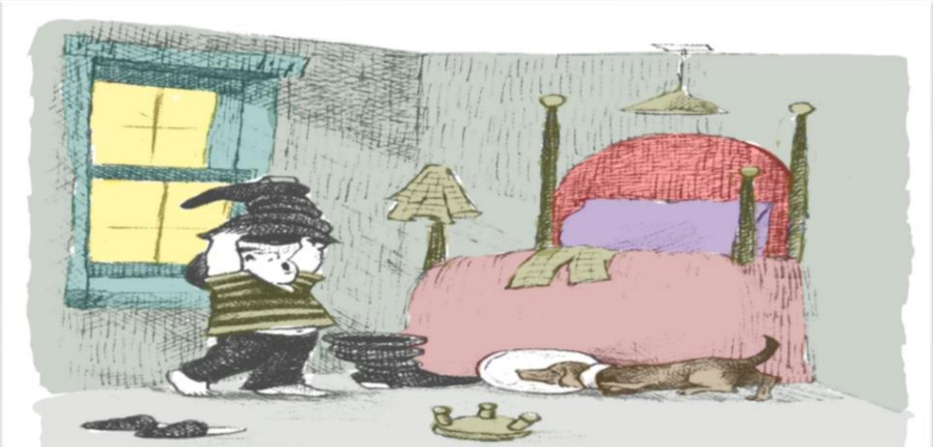

4

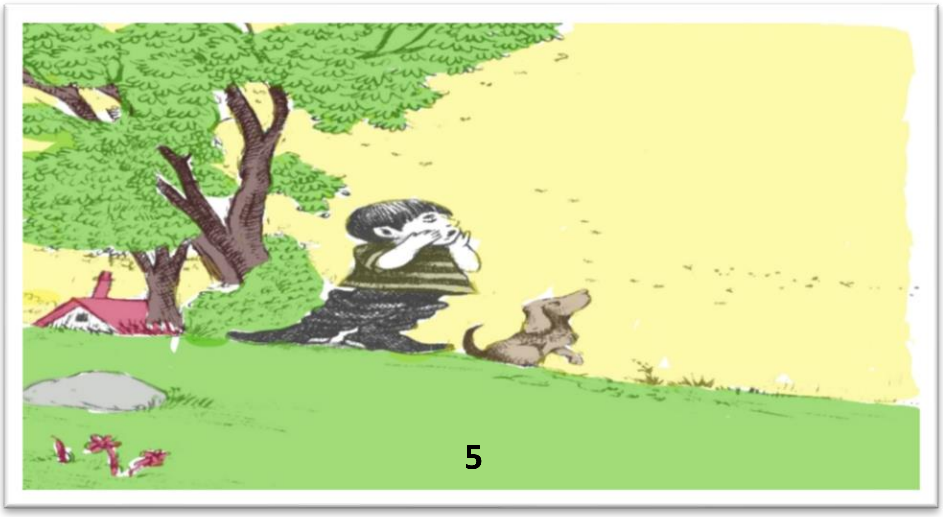

5

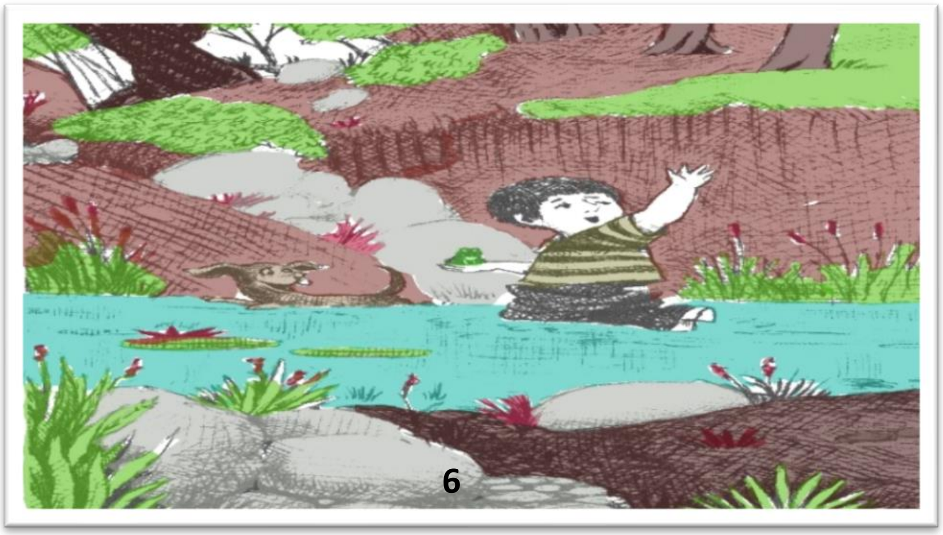

6
